# The Toxicity Differences of Fluralaner against the Red Imported Fire Ant (*Solenopsis invicta*) at Different Developmental Stages

**DOI:** 10.3390/ijms242115627

**Published:** 2023-10-26

**Authors:** Leyi Shao, Wei Wang, Xin Gong, Yinghao Yu, Junao Xue, Xinnian Zeng, Jiali Liu

**Affiliations:** College of Plant Protection, South China Agricultural University, Guangzhou 510642, China; leyishao2023@163.com (L.S.); 18819465384@163.com (W.W.); gongx@stu.scau.edu.cn (X.G.); 18879576581@163.com (Y.Y.); 15505487338@163.com (J.X.); zengxn@scau.edu.cn (X.Z.)

**Keywords:** contacting activity, fluralaner stress, invasive pest, metabolome, tolerance

## Abstract

The red imported fire ant (RIFA), *Solenopsis invicta*, is an invasive pest that causes damage to agricultural and ecological environments worldwide. Fluralaner is a new isoxazoline pesticide with the potential to become a control agent against RIFA. However, it is not clear whether *S. invicta* responds the same way to fluralaner at different reproductive stages. The present study firstly evaluated the toxicity of fluralaner to *S. invicta* at different developmental stages, finding that fourth instar larvae (LD_50_, 1744.23 mg/kg) and worker ants (LD_50_, 8.62 mg/kg) were differently susceptible to fluralaner, while the mortality rate of fourth instar larvae was significantly lower at the same concentration of 10 mg/L (5.56 ± 3.14%) than that of worker ants (62.22 ± 3.14%), demonstrating a greater tolerance to fluralaner. Subsequently, the metabolic responses of worker and larval ants to fluralaner stress (10 mg/L) were investigated using non-targeted metabolomics, which indicated that the amount of differential metabolites and the KEGG metabolic pathways enriched were different between workers and larvae when exposed to the same dose (10 mg/L) of fluralaner. Differential metabolites of larvae and worker ants under fluralaner stress were mainly concentrated in organic acids and their derivatives, lipids and lipid-like molecules, nucleosides, nucleotides, and analogues, combined with the enriched metabolic pathways, revealed that the differential metabolic responses of larvae and worker ants were mainly in energy metabolism, detoxification metabolism, and neurotransmitter ligands. Workers consumed more substrates in the arginine synthesis pathway (l-glutamic acid, l-aspartic acid, and fumaric acid) to provide energy for the detoxification (glutathione) of pesticides when exposed to fluralaner stress, and the high accumulation of l-aspartic acid induced excitotoxicity in the worker ants. Larval ants consumed more arachidonic acid to synthesize PG D2, and changes in the metabolism of antioxidants such as catechins, hesperidin, and l-ascorbic acid suggested that larvae were more capable of scavenging the ROS response than worker ants. The results of non-targeted metabolomics successfully revealed differences in the sensitivity of larvae and workers to fluralaner agents, providing insights into the fluralaner control of *Solenopsis invicta*.

## 1. Introduction

The red imported fire ant (RIFA), *Solenopsis invicta* (Hymenoptera: Formicidae), is a notorious invader worldwide due to its threat to native ants and arthropods, and the damage it causes to agriculture and to the ecological environment of the invaded area [1]. As a social insect, the RIFA nest members display elaborate task specialization, especially the workers, who occupy the majority of the nest individuals and display task-differentiation, such as in food searching, guarding, and nursing [2,3,4]. After the workers move food back to the nest, fourth instar larvae are responsible for digesting solid food into liquid. Later, nursing workers deliver the liquid food to other members in the colony [5]. Based on the vital role of workers and fourth instar larvae in the food supply, they are the main target in pesticide development. Moreover, it is necessary to know the toxicity of a certain active ingredient to these two different individuals. Currently, the main pesticide formulations for controlling RIFA are dustable powder and bait, the dust predominantly uses contact insecticides for mound and surface treatments, and the bait commonly uses stomach insecticides for controlling the whole colony [6]. However, by delaying the knockdown efficiency of the dust, the workers could carry the dust into the nest and transfer it to other members in the colony, which can also eliminate the whole colony. This new idea should evaluate the contact toxicity of the active ingredient in the dust to different castes and developmental stages.

Fluralaner, a novel isoxazoline (IRAC group 30) insecticide and acaricide, is currently marketed as Bravecto to control flea and tick infestation in animals [7,8]. Its target site is the glutamate- and γ-aminobutyric-acid-gated (GABA) chloride channels, which is different from carbamates, pyrethroids, neonicotinoids, and amidinohydrazone (mainly Hydramethylnon). Fluralaner even has a unique binding site which is different from that of fipronil on the GABA receptor, showing high insecticidal activity against fipronil-resistant insects [9,10]. The high activity of fluralaner against multiple agricultural pests has been explored, e.g., *Laodelphax striatellus*, *Tetranychus urticae* [9], *Chilo suppressalis* [11], *Spodoptera litura* [12], *Henosepilachna vigintioctopunctata*, *Megalurothrips usitatus*, and *Phyllotreta striolata* [13], suggesting its potential to be developed as an agricultural insecticide. Moreover, this chemical has been proven to have low toxicity to zebrafish [14]. Thus, fluralaner can be an excellent candidate for controlling RIFA nesting near fish ponds because of its safety to aquatic animals.

In previous studies, RIFA workers and larvae showed significant differences in fipronil toxicity. Cytochrome P450 was considered responsible for the difference because its activity was 24-fold higher in larvae than in workers [15]. It is still unclear whether the fluralaner response between workers and larvae is different. In this study, we first investigated the toxicity of fluralaner against different developmental stages of *S. invicta*. To understand the molecular mechanism and metabolic pathway of *S. invicta* response to fluralaner, the metabolome of control and fluralaner-treated *S. invicta* were identified. These results will provide more theoretical information for developing fluralaner as a pesticide to control *S. invicta*.

## 2. Results

### 2.1. Topical Toxicity of Fluralaner against S. incivta

The results of the contact toxicity of fluralaner against *S. incivta* at different reproductive stages of red fire ants are shown in Table 1. It can be seen that the differences in toxicity between workers and larvae were significant, with the LD_50_ values of larvae being much higher than those of adults, which was even 202.35 times higher than that of adults. Meanwhile, it is obvious from Figure S1 that the mortality rate of adults (62.22 ± 3.14%) was significantly higher than that of larvae (5.56 ± 3.14%) under the pressure of 10 mg/L of fluralaner. Both from the point of view of the LD_50_ values of larvae and adults, as well as the mortality rates of larvae and adults under the same concentration conditions, a significant difference in the sensitivity to the agent between fourth instar larvae and adults can be illustrated, with larvae showing a stronger tolerance to fluralaner than adults.

### 2.2. Effect of Fluralaner on the Metabolites of S. incivta at Different Reproductive Stages

**Figure 1:**
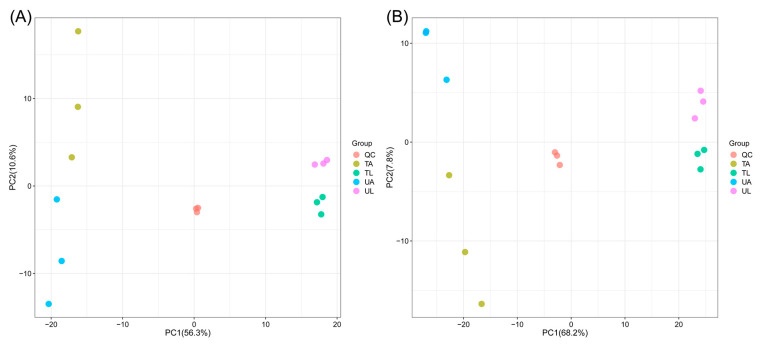

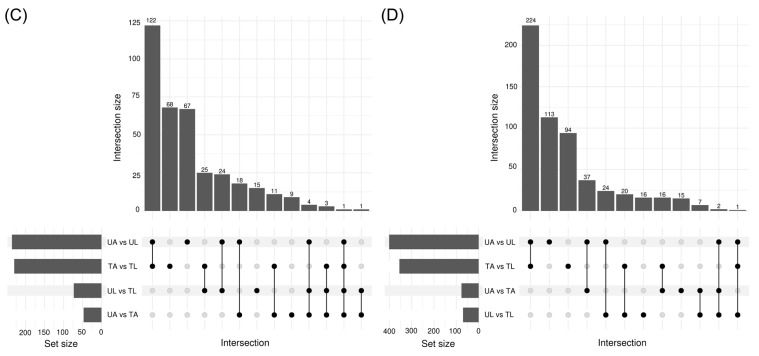
PCA and Upset Venn analysis of DEMs. (**A**) (PCA) and (**C**) (Upset Venn) means anionic mode, (**B**) (PCA) and (**D**) (Upset Venn) means cationic mode.

**Figure 2:**
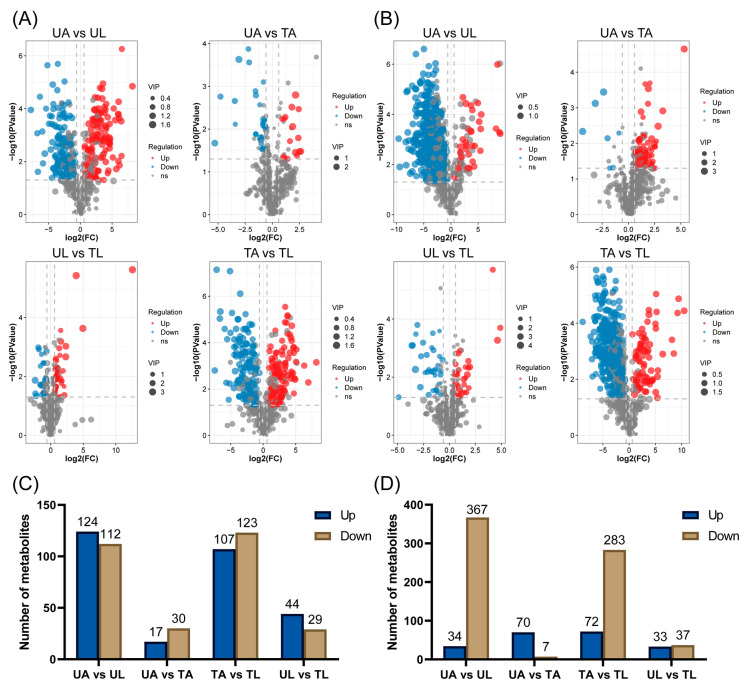
Upward and downward changes in metabolites. (**A**) (Volcanic composition) and (**C**) (Number of metabolites) means anionic mode, (**B**) (Volcanic composition) and (**D**) (Number of metabolites) means cationic mode.

The results of principal component analysis (PCA) showed that these samples were effectively separated in both anionic and cationic modes, with a good stability of the QC samples (Figure 1A,B), which confirms that the metabolomic data were well analyzed. The metabolite analysis identified a total of 368 differentially expressed metabolites in the anionic mode (DEMs, VIP > 1; *p* < 0.05, *n* = 3). In UA vs. UL, UA vs. TA, UL vs. TL, and TA vs. TL, 236, 47, 230, and 73 DEMs were found, respectively (Figure 1C and Figure S2). In contrast, 569 DEMs were analyzed in the cationic mode; DEMs in the four different groups of UA vs. UL, UA vs. TA, UL vs. TL, and TA vs. TL were 401, 77, 355, and 70 (Figure 1D and Figure S2). It was obvious from Figure 2 that the number of DEMs varied among the four comparison groups, while both identical and DEMs existed in the different comparison groups. Among these four comparative groups of DEMs, the present study focused on two groups, UA vs. TA and UL vs. TL. The number of DEMs up-regulated by UA vs. TA and down-regulated by UL vs. TL in the anionic mode was 17 and 107, respectively, while the number of DEMs down-regulated by UA vs. TA was 30, 123. The number of DEMs up-regulated by UA vs. TA (70) and those up-regulated by UL vs. TL (72) in the cationic mode were close to each other, whereas the number of DEMs down-regulated by UA vs. TA (7) was less than that down-regulated by UL vs. TL (283). This suggested that larval ants produced more drastic changes in metabolites than worker ants after fluralaner treatment. The UA vs. TA and UL vs. TL groups shared 9 identical DEMs in different assay modes. The UA vs. TA group had 9 (anionic mode) and 15 (cationic mode) DEMs in different modes, while the UL vs. TL group scored 15 (anionic mode) and 16 (cationic mode) DEMs, respectively. It is suggested that the response metabolites of larval ants to fluralaner contain both the same metabolites as those of adult workers as well as their respective specific response metabolites.

### 2.3. Classes of DEMs

**Figure 3:**
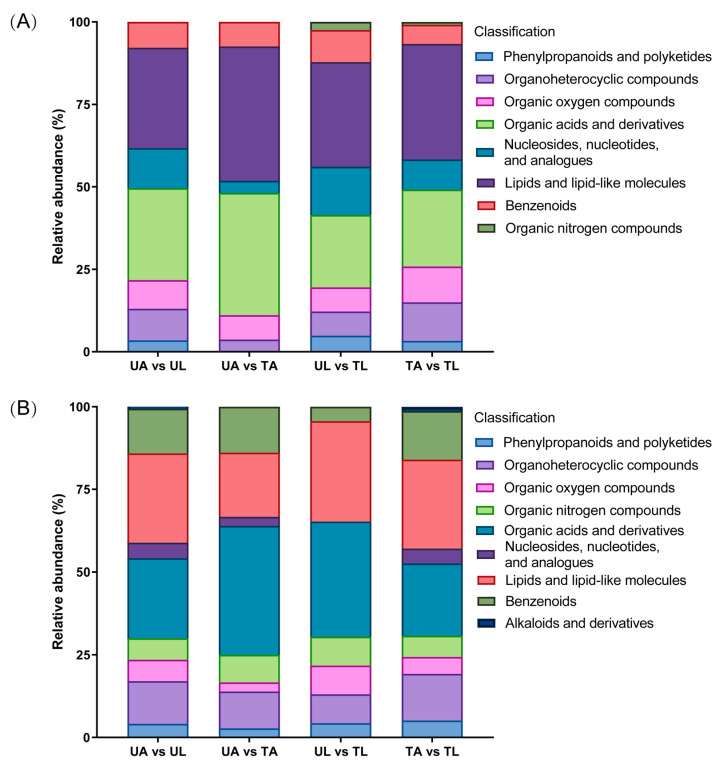
Classifications of DEMs with four pairwise comparisons. (**A**) means anionic mode, (**B**) means cationic mode.

An analysis of the relative abundance of DEMs in the different comparison groups suggested that the fluralaner treatment mainly affected the biosynthesis of amino acids, fatty acid metabolism, and secondary metabolites in larvae and worker ants (Figure 3 and Figure S3). The cationic mode identified one more class of alkaloids and derivatives than the anionic mode, which occurred only in the UA vs. UL (vindoline) and TA vs. TL (piperine and ecgonine) groups. UA vs. TA annotated six and eight metabolite classes in anionic and cationic modes, respectively, while UL vs. TL annotated eight (anionic mode) and seven (cationic mode) metabolite classes. It indicated that the types of metabolites produced by larvae in the presence of halothane differed from those of adults. In addition, in the cationic mode, the DEMs in the UA vs. TA groups were mainly annotated to phenylpropanoids and polyketides, organoheterocyclic compounds, organic oxygen compounds, organic nitrogen compounds, organic acids and derivatives, nucleosides, nucleotides, and analogues, lipids, and lipid-like molecules and benzenoids, suggesting that these metabolites were significantly changed after fluralaner treatment. However, UL vs. TL contained a higher proportion of phenylpropanoids and polyketides, organic oxygen compounds, organic nitrogen compounds, lipids, and lipid-like molecules compared to UA vs. TA. The anionic mode was similar for organoheterocyclic compounds, nucleosides, nucleotides, analogues, and benzenoids.

### 2.4. KEGG Pathway Enrichment Analysis

**Figure 4:**
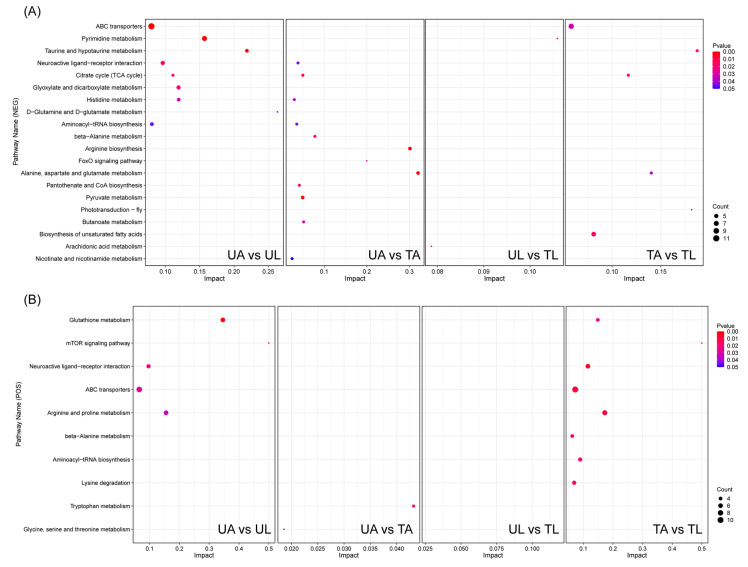
KEGG functional pathway significantly enriched for metabolites. (**A**) means the KEGG pathway enriched for DEMs in anionic mode, (**B**) means cationic. *p* < 0.05.

Figure S4 displayed the KEGG pathways enriched to DEMs in all comparison groups, as can be seen in 20 and 15 metabolic pathways in anionic and cationic modes, respectively. In the anionic mode, 9, 12, 2, and 6 pathways with *p*-value < 0.05 were found in UA vs. UL, UA vs. TA, UL vs. TL, and TA vs. TL, respectively (Figure 4A). Among these pathways, ABC transporters, taurine and hypotaurine metabolism, and citrate cycle (TCA cycle) were the common pathways in UA vs. UL and TA vs. TL, with the TCA cycle also present in UA vs. TA. Neuroactive ligand-receptor interaction, histidine metabolism, and aminoacyl-tRNA biosynthesis were the common pathways in UA vs. UL and UA vs. TA. Pantothenate and CoA biosynthesis were the common pathways in UA vs. TA and TA vs. TL. Beta-alanine metabolism, arginine biosynthesis, foxO signaling pathway, pantothenate and CoA biosynthesis, pyruvate metabolism, butanoate metabolism, and nicotinate and nicotinamide metabolism were unique to UA vs. TA, whereas pyrimidine metabolism and arachidonic acid metabolism were exclusive to UL vs. TL. In the cationic mode, 5, 2, and 8 pathways with *p*-value < 0.05 were found in UA vs. UL, UA vs. TA, and TA vs. TL, respectively; no pathways with *p*-value < 0.05 were found in UL vs. TL (Figure 4B). All five of the following—glutathione metabolism, mTOR signaling pathway, neuroactive ligand-receptor interaction, ABC transporters, and arginine and proline metabolism—were present in UA vs. UL and TA vs. TL. Tryptophan metabolism and glycine, and serine and threonine metabolism were specific in UA vs. TA. Beta-alanine metabolism, aminoacyl-tRNA biosynthesis, and lysine degradation were only enriched in TA vs. TL. The results of KEGG-enriched metabolic pathways could illustrate that the metabolic pathways of larvae and adults were different under normal conditions, as well as the metabolic pathways stimulated by larvae being different from those of adults after fluralaner treatment.

### 2.5. Expression of DEMs

**Figure 5:**
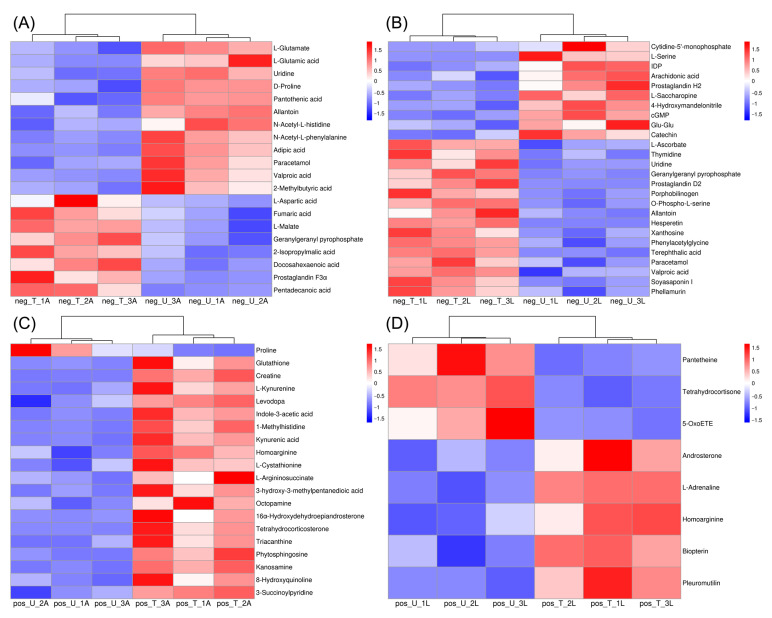
Heat map of metabolite hierarchical clustering for significant enrichment of KEGG pathway in *S. invicta* at different developmental stages. (**A**,**B**) means anionic mode, (**C**,**D**) means cationic mode.

The heatmaps based on the KEGG database annotations showed the distribution and expression of DEMs in different comparison groups (Figure 5 and Figure S5). The current study focused on the DEMs between UA vs. TA and UL vs. TL. It can be seen that a total of 20 and 26 DEMs were identified in the UA vs. TA and UL vs. TL groups, 12 metabolites were down-regulated, and 8 metabolites were up-regulated in TA compared to UA, while 10 metabolites were down-regulated and 26 metabolites were up-regulated in TL compared to UL. On the other hand, a total of 20 and 8 DEMs were identified in the UA vs. TA and UL vs. TL groups, respectively, with 3 metabolites down-regulated and 5 metabolites up-regulated in TA compared with UA, and 71 metabolites down-regulated and 19 metabolites up-regulated in TL compared with UL. There were 6 identical DEMs for UA vs. TA and UL vs. TL in both anionic and cationic modes’ in addition to the upregulation of both geranylgeranyl pyrophosphate and homoarginine; the remaining 4 DEMs did not change in the same way (Figure 5).

In order to screen potential DEMs in different comparison groups more precisely, KEGG and HMDB databases were further combined to obtain the results shown in Table 2. The DEMs identified in the anionic and cationic modes were categorized into eight classes. The most abundant metabolite types of DEMs in the UA vs. TA group were organic acids and derivatives, lipids, and lipid-like molecules, while, in the UL vs. TL group, the most abundant metabolite types of DEMs were lipids and lipid-like molecules, organic acids and derivatives, and nucleosides, nucleotides, and analogues. Compared to the UA vs. TA group, the UL vs. TL group contained more phenylpropanoids and polyketides, nucleosides, nucleotides, and analogues and benzenoids. Among these DEMs, homoarginine (organic acids and derivatives), allantoin (organoheterocyclic compounds), uridine (nucleosides, nucleotides, and analogues), geranylgeranyl pyrophosphate, valproic acid (lipids and lipid-like molecules), and paracetamol (Benzenoids) were common DEMs in the UA vs. TA and UL vs. TL groups, while the remaining DEMs were not the same in both comparison groups. In particular, phenylpropanoids and polyketides-like metabolites (hesperetin and catechin) were present only in the UL vs. TL group, and organic oxygen compound-like metabolites (pantothenic acid and l-Kynurenine) were found exclusively in the UA vs. TA group. In addition, the results in the table reflected the differences in the up- or down-regulation of metabolites by TA (relative to UA) and TL (relative to UL). All these results indicated that the metabolic response of larval ants to fluralaner was different from that of adult workers to fluralaner.

## 3. Discussion

Fluralaner is safer to non-target organisms and has delayed toxicity to pests compared to fipronil, which is important for the control of swarming pests and the safety of the environment [14,16]. Previous studies have focused more on aspects such as the toxicity of fluralaner to insects or environmental safety [11], with less studies reporting the metabolic response of *S. incivta* to fluralaner. Fluralaner is more toxic than fipronil to small brown planthoppers (*Laodelphax striatellus*) and the fruit fly (*Drosophila melanogaster*); fluralaner also exhibited superior feeding and contact toxicity against *S. incivta* workers compared to other isoxazoline pesticides such as benzoates, benzyl-benzoate, and n-pentyl benzoate [9,17,18]. In addition, the good horizontal transfer of fluralaner among *S. incivta* workers indicates its great potential as an agent for controlling *S. incivta* or other social pests [19]. Considering the differences in food availability for fourth instar larvae and workers in *S. incivta*, it is particularly necessary to understand the differences in toxicity of fluralaner between the two different insect states. A significant difference was found between the LD_50_ values of the fourth instar larvae and workers of the red fire ant *S. incivta* treated with fluralaner, with the LD_50_ values of the larvae being more than 200 times higher than those of adults. At the same concentration of 10 mg/L, the mortality rate of adults was significantly higher than larvae, which suggested that workers of the red fire ant *S. incivta* were more sensitive to fluralaner than fourth instar larvae. The further results of metabolites demonstrated the metabolic response to fluralaner was different in the UA vs. TA and UL vs. TL groups in the positive and negative ion mode. Among these DEMs, in addition to the presence of six identical DEMs such as allantoin, uridine, geranylgeranyl pyrophosphate, valproic acid, paracetamol, and homoarginine, a large number of different DEMs were also present. These DEMs were mainly clustered in organic acids and derivatives, lipids, and lipid-like molecules, affecting the metabolic responses of larval and adult workers to fluralaner from energy metabolism, detoxification metabolism, and neurotransmitter-ligand aspects. The results of the changes in the metabolites of *S. incivta* by mass spectrometry imaging and untargeted metabolomics by Du et al. [20] revealed significant changes in carbohydrates, amino acids, pyrimidines, and their derivatives in *S. incivta* workers at 48 h of indoxacarb treatment, especially a significant decrease in uracil content in treated workers, which is similar to the changes in uridine in workers in this study. Pyrimidine metabolism is essential for maintaining basic cellular functions in organisms, such as DNA and RNA biosynthesis, while its occurrence in mammalian organisms is often accompanied by cancer stress [21]. However, uridine and thymidine metabolites in the metabolic pathway of pyrimidine metabolism were significantly up-regulated in fluralaner-treated larvae, suggesting that fluralaner would have a greater effect on pyrimidine metabolism in larvae than in adults, and whether larval mortality is related to abnormalities in pyrimidine metabolism remains to be investigated in depth.

Similar to fipronil, the *P450* gene in workers may play a role in the detoxification metabolism of fluralaner, but not in the detoxification of glutathione S-transferase (GST) [19]. In this study, a significant up-regulation of glutathione metabolites was found to occur in workers, the main reason for this difference might be the difference in the concentration of fluralaner used to treat the insects, which was previously reported to be at LC_30_, while, in the present study, it was at a concentration of 10 mg/L (the mortality rate of the workers reached 62.22 ± 3.14%). A further exploration of the molecular mechanisms underlying the response of *S. incivta* at different developmental stages to fluralaner through the transcriptome is still needed in the future.

Previous studies showed that insects activate energy metabolic pathways to provide energy for pesticide detoxification when exposed to pesticides [22]. Amino acids are important components of all living cells and can be used as a source of energy [23], which can be categorized into three groups, acidic amino acids, basic amino acids, and neutral amino acids, based on the number of amino (-NH_2_) and carboxylic (-COOH) groups [24]. l-Malate, fumaric acid, and 2-isopropylmalic acid are related to the citrate cycle (TCA cycle) and pyruvate metabolism, and these types of metabolisms are closely related to energy metabolism. Compared to untreated adult workers, decreased l-Glutamic acid and elevated l-Aspartic acid and fumaric acid levels in fluralaner-treated adults activated the arginine biosynthesis pathway, which may positively affect energy utilization and muscle contraction by stimulating adults to consume more energy [25,26,27,28]. Homoarginine is a methylene homologue of arginine, and both are similar in nature, with the main difference being the number of methylene groups (-CH_3_) in the main chain, while both can have a similar effect on the utilization of energy in organisms [29,30]. In this study, it was found that the l-Aspartic acid, fumaric acid, and homoarginine content in the arginine metabolic pathway were significantly up-regulated in the UA vs. TA group, whereas in the UL vs. TL group, only the homoarginine content was up-regulated. All these results could indicate that adults activated more energy metabolic pathways to provide energy for pesticide detoxification than larvae.

Typically, the use of pesticides can cause individual insects to produce large amounts of reactive oxygen species (ROS), which can lead to individual DNA damage and apoptosis [31]. To avoid the toxic effects of ammonia produced during the catabolism of proteins and nucleic acids in insects, the compound uric acid is synthesized in the system in response to these toxic effects. Uric acid is further metabolized to allantoin in some insects [32]. Although uric acid was not detected in the results of DEMs, allantoin levels were elevated in fluralaner-treated larvae compared to untreated larvae, suggesting that uric acid may be produced in their bodies in response to the toxic effects of the catabolism of proteins or nucleic acids in their bodies. Meanwhile, the large up-regulation of nucleosides, nucleotides, and analogues metabolites in fluralaner-treated larvae also proves this point. The isoprenoid biosynthesis pathway plays an important role in cellular activities and can produce a large number of biologically active metabolites. The geranylgeranyl pyrophosphate synthesized in this pathway is a substrate for the synthesis of long-chain isoprenoids as well as a precursor for the synthesis of many molecules essential for cellular functions, such as cholesterol, heme A, ubiquinone, ethanol, and fasolactones [33]. It is also essential for cell survival in cell signaling pathways where geranylgeranyl pyrophosphate is used to modify GTPases such as Ras [34]. The levels of geranylgeranyl pyrophosphate were significantly up-regulated in both UA vs. TA and UL vs. TL groups in the study, suggesting that both larval and adult workers respond to the effects of fluralaner by up-regulating geranylgeranyl pyrophosphate.

Most insects exposed to pesticides undergo significant changes in their own antioxidant defense enzymes like catalase (CAT), superoxide dismutase (SOD), glutathione reductase (GR), glutathione s-transferase (GST), and glutathione (GSH) to maintain cellular redox homeostasis [35,36]. Ascorbic acid is the major cellular antioxidant that can alter the transport and metabolic mechanisms of the dehydroascorbic acid cycle; moreover, the ascorbate-glutathione cycle is an important system for clear reactive oxygen species (ROS) [37,38,39]. In addition, catechins and hesperidin are well known antioxidants that can stimulate the antioxidant defense system of insects to repair ROS damage induced by pesticide stress [40]. The significantly decreased levels of catechins and increased levels of hesperetin in the fluralaner-treated larvae may have stimulated the antioxidant defense system of the larvae to scavenge ROS. However, no significant changes in these two metabolites were observed in the UA vs. TA group. Glutathione was significantly upregulated in TA compared to UA, whereas no significant changes in glutathione were found in UL vs. TL. Similarly, l-Ascorbate was significantly upregulated in TL compared to UL, whereas no significant change in l-Ascorbate was found in UA vs. TA. This indicated that the antioxidant defense system of both larvae and adults could be activated under the treatment of fluralaner to enhance the individual’s ability to scavenge ROS, while the main substances that caused the enhancement of ROS scavenging ability in larvae and adults were different. Arachidonic acid can help alleviate oxidative stress in insects and has a positive effect on scavenging ROS, however, it can also cause inflammation and larval mortality when increased [41].

Metabolic and immune responses in insects are closely linked, while the mechanisms involved are unclear [42,43]. Tryptophan metabolism is an important physiological metabolism in organisms and can be involved in important immune functions [44]. The l-Kynurenine, indole-3-acetic acid, and kynurenic acid content in the tryptophan metabolism of fluralaner-treated adults rose compared to untreated adults, perhaps stimulating tryptophan metabolic pathways in response to the effects of fluralaner. Glycine, tryptophan, and threonine metabolism often co-occur in organisms, producing precursors of many low-molecular-weight key metabolites such as creatine, glutathione, heme, purines, and porphyrins, which play important roles in metabolic regulation, antioxidant responses, and neural function [45,46]. The significant increase in creatine, l-Cystathionine, and glutathione content in fluralaner-treated adults compared to untreated adults could indicate that fluralaner-stimulated adult workers activated glycine, tryptophan, and threonine metabolic pathways in the organism, which induced their own defense regulatory functions.

As acidic amino acids, glutamate and aspartate are both major excitatory neurotransmitters in the central nervous system and can induce excitotoxicity in individuals when exposed to nerve agents [47,48]. Larval ants did not show significant changes in glutamate and aspartate content before and after fluralaner treatment. While the l-Glutamate content was reduced and l-Aspartic acid content was increased in fluralaner-treated adult workers compared to untreated adult workers, the high accumulation of l-Aspartic acid content can also lead to excitotoxicity in adult workers.

Cyclooxygenase (COX) mediates the formation of prostaglandins during the oxygenation of arachidonic acid, among which Prostaglandin H2 (PGH2) is the direct COX product of arachidonic acid, after which PGH2 undergoes an enzymatic reaction that converts it into the major effectors prostaglandin, prostaglandin F2α, prostaglandin E2, prostaglandin D2, and thromboxane A2 [49]. The role and function of PGs as an autocrine or paracrine signal has been more extensively studied in mammals, mediating a variety of physiological processes in the body such as sleep, pain, immunity, and metabolism [50]. Recent studies have revealed that PGs (PGA2, PGD2, PGE2, PGF2α, and PGI2) are not exclusive to mammals and are successively being detected in insect species [51], of which PG A2, PG D2, PG E2, and PG F2α have been reported to be synthesized during microsomal preparations of larval fat bodies and blood cells [52,53]. As in mammals, PGs also play important functions in regulating various physiological processes in insects similar to those similar in mammals, such as influencing egg-laying behavior [54] and mediating cellular immune responses [55]. Some studies revealed that the biosynthetic pathways of PGs in insects and mammals significantly differ due to the lack of homologs, and only the synthesizing factors of PG E2 and PG D2 were currently investigated [54,56]. Three metabolites in the arachidonic acid pathway, arachidonic acid, PG H2, and PG D2, were detected in the DEMs of UL vs. TL, in which the levels of arachidonic acid and PG H2 were significantly decreased in fluralaner-treated larval ants, whereas PG D2 was significantly up-regulated, suggesting that larval ants, compared to adult workers, when subjected to fluralaner, consume more arachidonic acid for the synthesis of PG D2 to cope with the effects of pharmaceutical stress.

## 4. Material and Methods

### 4.1. Insects and Chemicals

The red imported fire ant colonies were collected at a farm in Zengcheng, Guangzhou, Guangdong province, China (23°16′ N, 113°23′ E) from March to May in 2017. This farm belongs to South China Agricultural University and the staff make sure that the RIFA colonies were not treated with any pesticide. The social forms of collected RIFA colonies were polygyne which was identified following the method of Valles and Porter [57]. Ants were removed from nests using the water floating method and placed in rearing boxes (50 cm in length, 40 cm in width, and 30 cm in height) with Teflon coated on the inner walls. The colonies were reared with mealworm larvae *Tenebrio molitor* and water. Before the experiment, the colonies were kept in the laboratory for at least two weeks to acclimatize the laboratory environment. All experiments were conducted at 26 ± 1 °C and 60 ± 1% RH.

Fluralaner (99.87%) was purchased from MedChem Express (Shanghai, China). Acetone was purchased from Aladdin Chemical Co. (Shanghai, China). Other reagent was purchased from Thermo Fisher (Carlsbad, CA, USA).

### 4.2. Topical Toxicity

Topical toxicity of fluralaner against adult workers and 4th instar larva was conducted according to the methodology reported by Xiong et al. [15] with some improvements. Fluralaner was dissolved in acetone and prepared in five concentrations (Table S1) for use; ants treated with equivalent acetone were served as the control. Each treatment was replicated 3 times, with 30 insects per replication; test insects were similar in size and health status. On the day of the experiment, ants were collected into a plastic box (apply a small amount of talcum powder to the mouth of the bowl) with a brush from the rearing cage, and then knocked down with CO_2_. Knocked-down ants were individually treated with 1 μL of fluralaner solution topically to the pronotum using a Hand-Operated micro-applicator (Burkard Scientific Ltd., Rickpmanosworth, UK). The ants were held briefly for the treatment to dry before they were moved back to the plastic box. Treated and control ants were kept in boxes under laboratory condition (26 ± 1 °C, 60 ± 1% RH) with a photoperiod of 12:12 (L:D) h and were provided with suitable moisture and yellow mealworms for the test insects to remain healthy. The LD_50_ value was calculated based on the number of deaths observed for 24 h. Larvae were considered dead when their bodies shriveled up or turned black due to dehydration. Individual worker ants that were unable to stand, or in which fewer than 3 legs could be moved when the body was gently touched with a fine brush, were considered dead.

### 4.3. Metabolomic Data Acquisition and Processing

To find the metabolites related to fluralaner response, ants were treated with 10 mg/L (1 μL) fluralaner solution, the surviving ants were collected 24 h after treatment. In the control group, ants were treated with 1 μL acetone solution. A total of 4 subgroups were designed, UA (untreated adult worker ants), UL (untreated 4th instar larvae), TA (flutriafol-treated adult worker ants), and TL (flutriafol-treated 4th instar larvae). The two groups, UL vs. TL and UA vs. TA, were the focus of this study. The whole body (100 mg) was ground with liquid nitrogen and the homogenate was resuspended with prechilled 80% methanol using a well vortex. The samples were incubated on ice for 5 min and then centrifuged at 15,000× *g*, 4 °C for 20 min. The supernatant was diluted to a final concentration containing 53% methanol by LC-MS grade water and was recentrifuged at 15,000× *g*, 4°C for 20 min. Finally, the supernatant was injected into the LC-MS/MS system for analysis. Three biological replicates were used for each treatment. The extracts were analyzed using an Vanquish UHPLC coupled with an Orbitrap Q Exactive^TM^ HF mass spectrometer (Thermo Fisher Scientific, San Jose, CA, USA). Samples (2 μL) were injected onto a Hypersil Goldcolumn (100 × 2.1 mm, 1.9 μm) using a 17 mm linear gradient at a flow rate of 0.2 mL/min. The eluents for the positive polarity mode were eluent A (0.1% Formic Acid in Water) and eluent B (Methanol). The eluents for the negative polarity mode were eluent A (5 mM ammonium acetate, pH 9.0) and eluent B (Methanol).The solvent gradient was set as follows: 2% B, 1.5 min; 2–85% B, 3 min; 85–100% B, 10 min; 100–2% B, 10.1 min; 2% B, 12 min. Q ExactiveTM HF mass spectrometer was operated in positive/negative polarity mode with spray voltage of 3.5 kV, capillary temperature of 320 °C, sheath gas flow rate of 35 psi and aux gas flow rate of 10 L/min, S-lens RF level of 60, and aux gas heater temperature of 350 °C.

The raw data files generated using UHPLC-MS/MS were processed using the Compound Discoverer 3.3 (CD3.3, Thermo Fisher Scientific, San Jose, CA, USA) to perform peak alignment, peak picking, and quantitation for each metabolite. The main parameters were set as follows: peak area was corrected with the first quality control (QC), actual mass tolerance, 5 ppm; signal intensity tolerance, 30%; and minimum intensity. After that, peak intensities were normalized to the total spectral intensity. The normalized data were used to predict the molecular formula based on additive ions, molecular ion peaks, and fragment ions. And then peaks were matched with the mzCloud (https://www.mzcloud.org/ (accessed on 25 August 2023)), mzVault, and MassList database to obtain the accurate qualitative and relative quantitative results. Statistical analyses were performed using the statistical software R (R version R-3.4.3), Python (Python 2.7.6 version), and CentOS (CentOS release 6.6). When data were not normally distributed, they were standardized according to the following formula: sample raw quantitation value/(The sum of sample metabolite quantitation value/The sum of QC1 sample metabolite quantitation value) to obtain relative peak areas; compounds whose CVs of relative peak areas in QC samples were greater than 30% were removed, and, finally, the metabolites’ identification and relative quantification results were obtained. The metabolites were annotated using the KEGG database (https://www.genome.jp/kegg/pathway.html (accessed on 25 August 2023)) and HMDB database (https://hmdb.ca/metabolites (accessed on 25 August 2023)).

### 4.4. Statistical Analysis

Toxicity bioassays were analyzed using probit procedure in SPSS software (25.0 version, https://www.ibm.com/products/spss-statistics (accessed on 25 August 2023)).

Principal components analysis (PCA) was performed using Python (Python 3.5.0 version) and R (R version 3.4.3) to visualize the clustering among samples. We applied univariate analysis (*t*-test) to calculate the statistical significance (*p*-value). The metabolites with VIP (variable importance in the projection) > 1 and *p*-value < 0.05 and fold change (FC) ≥ 2 or FC ≤ 0.5 were considered to be differential metabolites (DEMs). The correlation between DEMs were analyzed using cor () in R language. Statistically significant correlations between DEMs were calculated using cor.mtest () in R language. *p*-value < 0.05 was considered as statistically significant and correlation plots were plotted using corrplot package in R language.

Volcano plots were used to filter metabolites of interest based on VIP, log2 (FoldChange), and -log10 (*p*-value) of metabolites using ggplot2 in R language.

For clustering heat maps, the data were normalized using z-scores of the intensity areas of DEMs and were plotted using Pheatmap package in R language.

The functions of these metabolites and metabolic pathways were studied using the KEGG database. The metabolic pathways enrichment of DEMs was performed. When ratios were satisfied by x/n > y/n, metabolic pathways were considered as enrichment, and when *p*-value of metabolic pathway < 0.05, metabolic pathway were considered as statistically significant enrichment.

## 5. Conclusions

In this study, the sensitivity of the fourth instar larvae and workers of *S. incivta* to fluralaner were different, showing that the larva displayed a greater tolerance to fluralaner than workers. Metabolomics analyses revealed that the metabolic responses of fourth instar larvae and workers to fluralaner were significantly different, although both were mainly involved in energy metabolism, detoxification metabolism, and neurotransmitter ligand metabolism. Adult workers consumed more of the substrates in the arginine synthesis pathway (l-Glutamate, l-Aspartate, and fumaric acid) to provide energy for the detoxification (glutathione) of the pesticide, and the high accumulation of l-Aspartate induced excitotoxicity in adults. Larval ants would consume more arachidonic acid to synthesize PG D2 in response to the effects of fluralaner, while their metabolic changes in antioxidants such as catechin, hesperidin, and l-Ascorbate suggested that larval ants were more capable of scavenging the ROS response under the influence of fluralaner than adult workers. To summarize, the present study provided new insights, from a metabolomic perspective, into the greater tolerance of the red fire ant *S. incivta* fourth instar larvae to fluralaner agents than adults.

## Figures and Tables

**Table 1:** Topical toxicity of fluralaner on different developmental stages of *S. invicta*.

Caste/Developmental Stage	Time (h)	LD_50_ (95% CL) (ng/Insect)	Weight (mg)	N	$\chi^{2}(\mathrm{df})$	*p*-Value
Adult worker	24	8.62 (7.75, 9.64)	1.06	540	28.08 (13)	0.242
Larva	24	1744.23 (1488.07, 2060.67)	1.10	540	21.71 (13)	0.594

CL: 95% confidence limits. *p*-values based on Chi-square goodness of fit test. *p*-values > 0.05 suggest the goodness of fit of the model.

**Table 2:** Potential DEMs between different comparison groups.

Class	DEMs (Neg)		DEMs (Pos)	
	UA vs. TA	UL vs. TL	UA vs. TA	UL vs. TL
Phenylpropanoids and polyketides	-	Hesperetin ^(↑)^; Catechin ^(↓)^	-	-
Organoheterocyclic compounds	Allantoin ^(↓)^	Allantoin ^(↑)^; l-Ascorbate ^(↑)^	Kynurenic acid ^(↑)^; Indole-3-acetic acid ^(↑)^	Biopterin ^(↑)^
Organic oxygen compounds	Pantothenic acid ^(↓)^	-	l-Kynurenine ^(↑)^	-
Organic nitrogen compounds	-	Porphobilinogen ^(↑)^	Phytosphingosine ^(↑)^	-
Organic acids and derivatives	*N*-Acetyl-l-phenylalanine ^(↓)^; l-Glutamate ^(↓)^; *N*-Acetyl-l-histidine ^(↓)^; l-Glutamic acid ^(↓)^; D-Proline ^(↓)^; l-Aspartic acid ^(↑)^; Fumaric acid ^(↑)^; l-Malate ^(↑)^	*O*-Phospho-l-serine ^(↑)^; l-Saccharopine ^(↓)^; l-Serine ^(↓)^; Phenylacetylglycine ^(↑)^; Glutamyl-glutamic acid ^(↓)^	Homoarginine ^(↑)^; Creatine ^(↑)^; 1-Methylhistidine ^(↑)^; 3-Succinoylpyridine ^(↑)^; l-Argininosuccinate ^(↑)^; l-Cystathionine ^(↑)^; Glutathione ^(↑)^; Levodopa ^(↑)^; Proline ^(↓)^	Homoarginine ^(↑)^; Pantetheine ^(↓)^
Nucleosides, nucleotides, and analogues	Uridine ^(↓)^	Uridine ^(↑)^; cGMP ^(↓)^; Xanthosine ^(↑)^; Thymidine ^(↑)^; Inosine diphosphate ^(↓)^; Cytidine-5’-monophosphate ^(↓)^	-	-
Lipids and lipid-like molecules	Geranylgeranyl pyrophosphate ^(↑)^; Valproic acid ^(↓)^; Pentadecanoic acid ^(↑)^; Adipic acid ^(↓)^; Prostaglandin F3α ^(↑)^; Docosahexaenoic acid ^(↑)^; 2-Methylbutyric acid ^(↓)^; 2-Isopropylmalic acid ^(↑)^	Geranylgeranyl pyrophosphate ^(↑)^; Valproic acid ^(↑)^; Soyasaponin I ^(↑)^; Prostaglandin D2 ^(↑)^; Phellamurin ^(↑)^; Prostaglandin H2 ^(↓)^; Arachidonic acid ^(↓)^	3-hydroxy-3-methylpentanedioic acid ^(↑)^; Tetrahydrocorticosterone ^(↑)^; 16α-Hydroxydehydroepiandrosterone ^(↑)^	Tetrahydrocortisone ^(↓)^; Androsterone ^(↑)^; 5-OxoETE ^(↓)^
Benzenoids	Paracetamol ^(↓)^	Paracetamol ^(↑)^; Terephthalic acid ^(↑)^; 4-Hydroxymandelonitrile ^(↓)^	Octopamine ^(↑)^	l-Adrenaline ^(↑)^

Note: “-” means no data, “^(↑)^” indicates up-regulation of metabolite expression in TL or TA compared to UL or UA, and “^(↓)^” indicates down-regulation.

## Data Availability

Data will be made available on request.

## Supplementary Materials

The supporting information can be downloaded at: https://www.mdpi.com/article/10.3390/ijms242115627/s1.
