# Supplementary material for: The Toxicity Differences of Fluralaner against the Red Imported Fire Ant (Solenopsis invicta) at Different Developmental Stages
Source: Int J Mol Sci. 2023 Oct 26;24(21):15627. doi: 10.3390/ijms242115627 (PMC10649654; doi:10.3390/ijms242115627)
Supplement: Supplementary file 1 [file ijms-24-15627-s001.zip › ijms-2660892-supplementary.pdf]

Table S1 Concentration gradient of topical toxicity of fluralaner to red fire ants at different developmental stages.

Developmental Stage	Fluralaner Concentration Gradient (mg/L)					
Adult	Acetone	1	3	6	9	12
Larva	Acetone	200	400	800	1600	3200

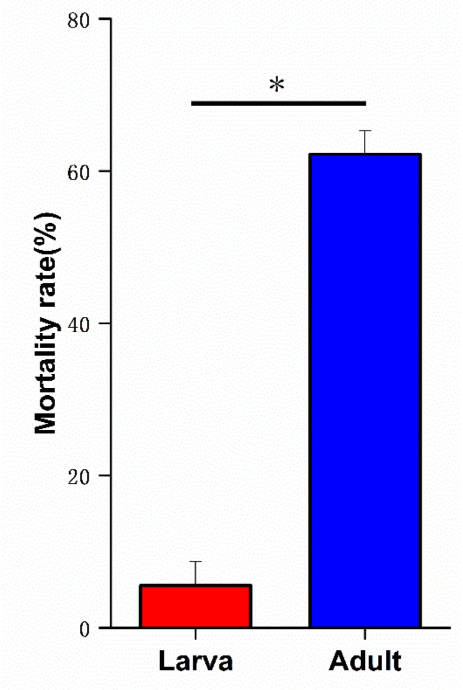

Figure S1. Mortality of *S. incivta* at different developmental stages under the same concentration (10 mg/L), * indicates significant differences by Student's *t* test (* $p < 0.05$).

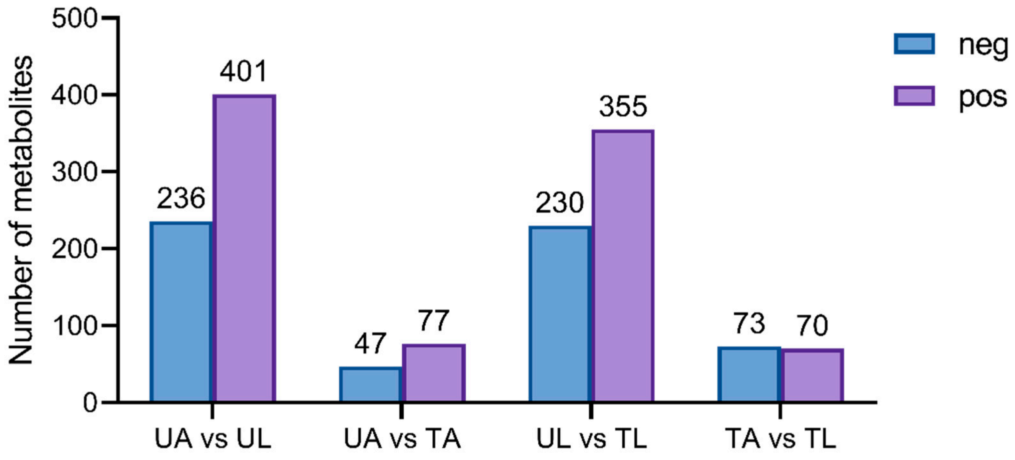

Figure S2. Number of DEMs in different comparison groups

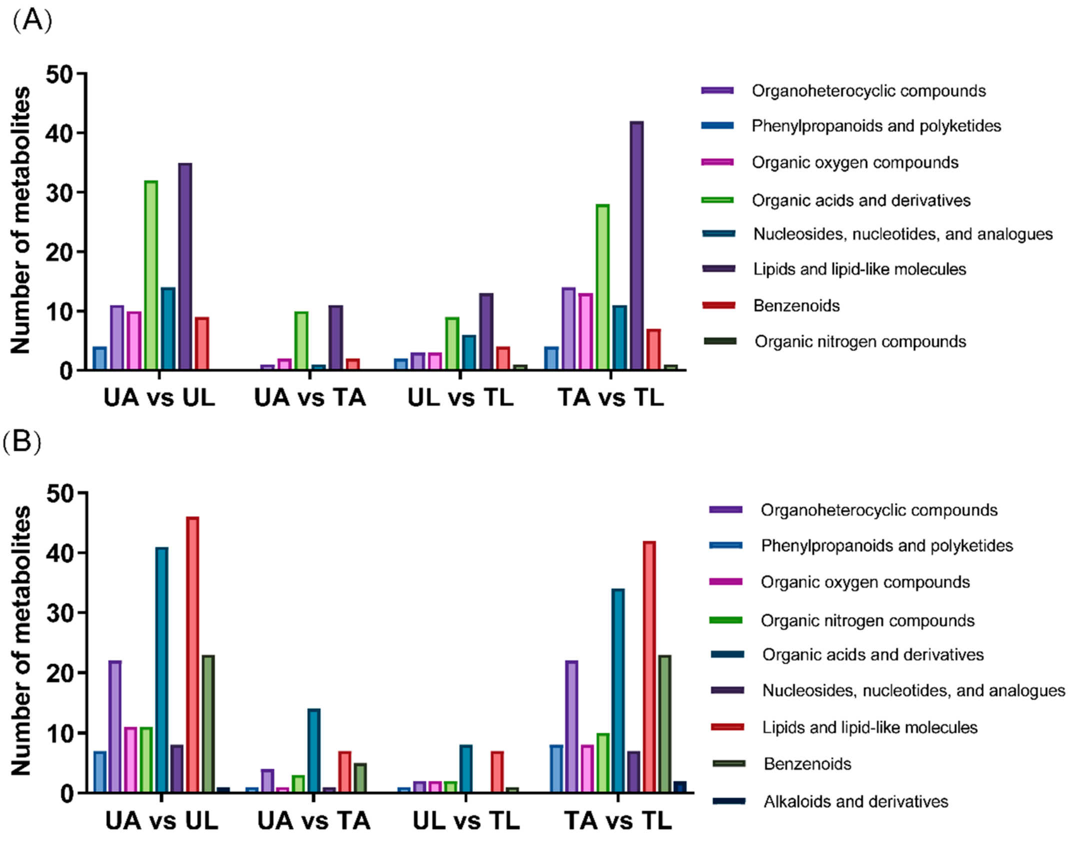

Figure S3. Comparison of classes of DEMs across comparison groups. (**A**) means anionic mode, (**B**) means cationic mode.

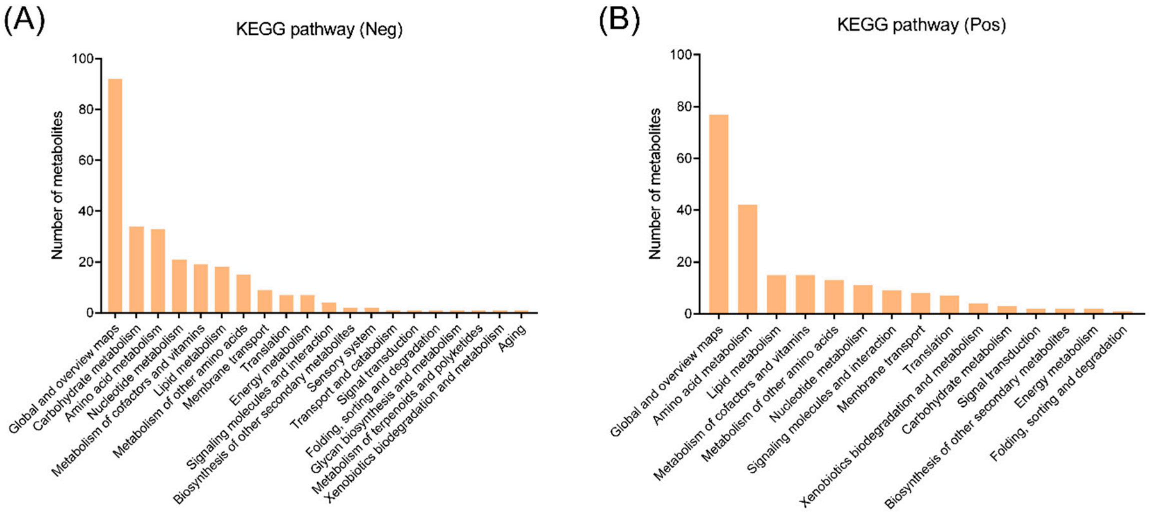

Figure S4. KEGG functional pathways for metabolite enrichment. From left to right, the number of metabolites in the column is ordered from highest to lowest. The higher the column, the more metabolites involved in the pathway among those identified. (**A**) means the KEGG pathway enriched for DEMs in anionic mode, (**B**) means cationic.

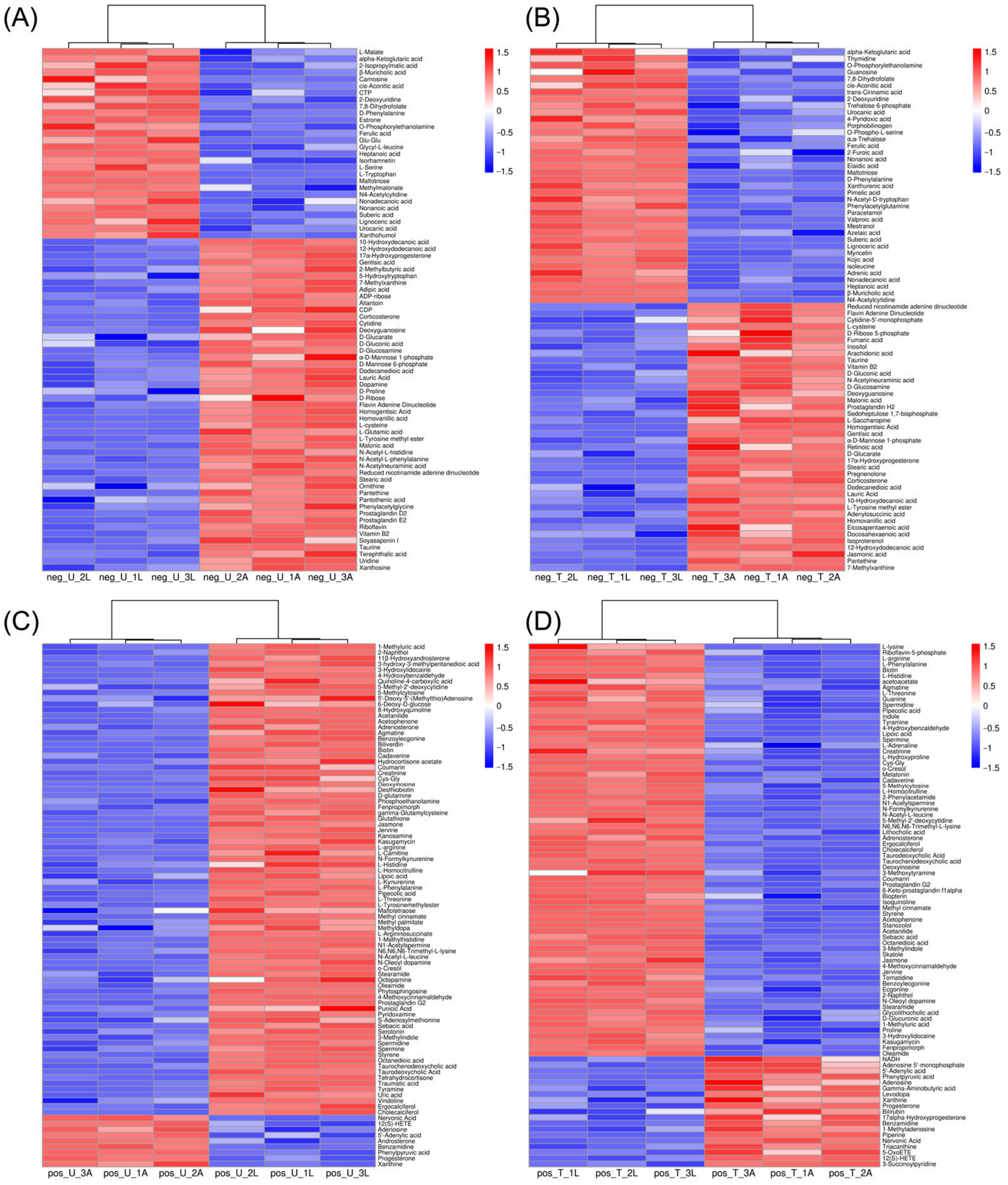

Figure S5. Heat map of metabolite hierarchical clustering of different classes of red fire ants significantly enriched in KEGG pathway. (**A**) and (**B**) means anionic mode, (**C**) and (**D**) means cationic mode.
